# Nonsteroidal antiinflammatory drug induced acute granulomatous interstitial nephritis

**DOI:** 10.1186/s13104-015-1788-2

**Published:** 2015-12-16

**Authors:** Jong Hwan Jung, Kyung Pyo Kang, Won Kim, Sung Kwang Park, Sik Lee

**Affiliations:** Department of Internal Medicine, Research Institute of Clinical Medicine of Chonbuk National University-Biomedical Research Institute of Chonbuk National University Hospital, Chonbuk National University Medical School, 634-18, Keum-Am Dong, Jeonju, 561-712 Republic of Korea

**Keywords:** Acute interstitial nephritis, Granulomatous inflammation, Nonsteroidal anti-inflammatory drugs

## Abstract

**Background:**

Acute interstitial nephritis is a common cause of acute kidney injury (AKI). The granulomatous inflammation is rarely but often manifests as a form of a granulomatous interstitial nephritis (GIN) in the kidney. Acute granulomatous interstitial nephritis is mainly associated with drugs, infection and autoimmune diseases.

**Case presentation:**

A 44-year-old-male visited our out-patient department with symptoms of nausea, vomiting, and general weakness that had developed over the previous 2 weeks. He had history of medication, nonsteroidal anti-inflammatory drugs. On admission to the general ward, his serum creatinine level was markedly elevated. GIN was confirmed by renal biopsy and 30 mg of corticosteroid per day was immediately initiated. Subsequently, his serum creatinine level and uremic symptoms dramatically decreased.

**Conclusion:**

Acute granulomatous interstitial nephritis is a rare but important disease on AKI. As long as we can carefully exclude infectious diseases as the cause of granulomatous lesion, acute granulomatous interstitial nephritis can be treated with steroid regardless of the etiologies. Since there is no proven treatment for the GIN yet, we can carefully suggest that moderate to high dosage corticosteroid can be helpful for prognosis in case of acute granulomatous interstitial nephritis of patients with AKI.

## Background

Acute interstitial nephritis (AIN) is a common cause of acute kidney injury (AKI) that is associated with several offending drugs. Drug-induced AIN mainly results from use of drugs such as antimicrobials, nonsteroidal anti-inflammatory drugs (NSAIDs), and anticonvulsants, and these drugs also may cause a granulomatous interstitial nephritis (GIN) less commonly [1]. In addition to these drugs, diverse etiologies including allergy, infection, allergic reaction and autoimmune disease can be other causes of AIN. A granulomatous inflammation is often clinically recognized as one form of chronic inflammation. However, GIN includes histological findings that are marked by the presence of granulomas with epitheloid histocytes; it can also be presented as an acute form [2]. According to a recent report, the most common cause of GIN is idiopathic. However, drugs, sarcoidosis, and tubulointerstitial nephritis and uveitis (TINU) could result in GIN [3]. Renal biopsy is the gold standard for diagnosis of AIN or unexplained AKI. Renal biopsy provides clinically useful information for the management of AKI. We describe an interesting case that was confirmed as GIN on renal biopsy.

## Case presentation

**Fig. 1:**
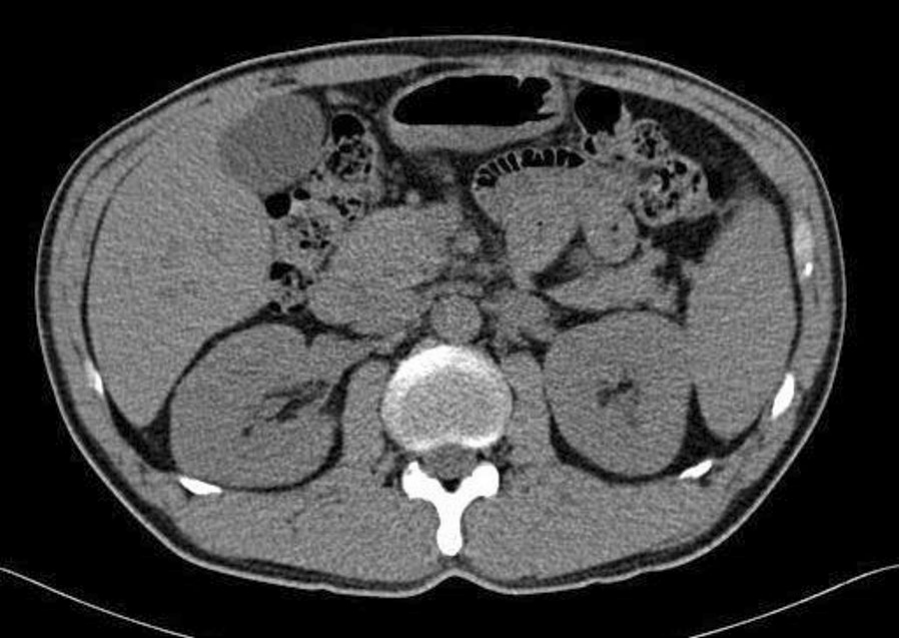
Non-contrast enhanced abdominal computed tomography showed both enlarged kidney and no hydronephrosis

**Fig. 2:**
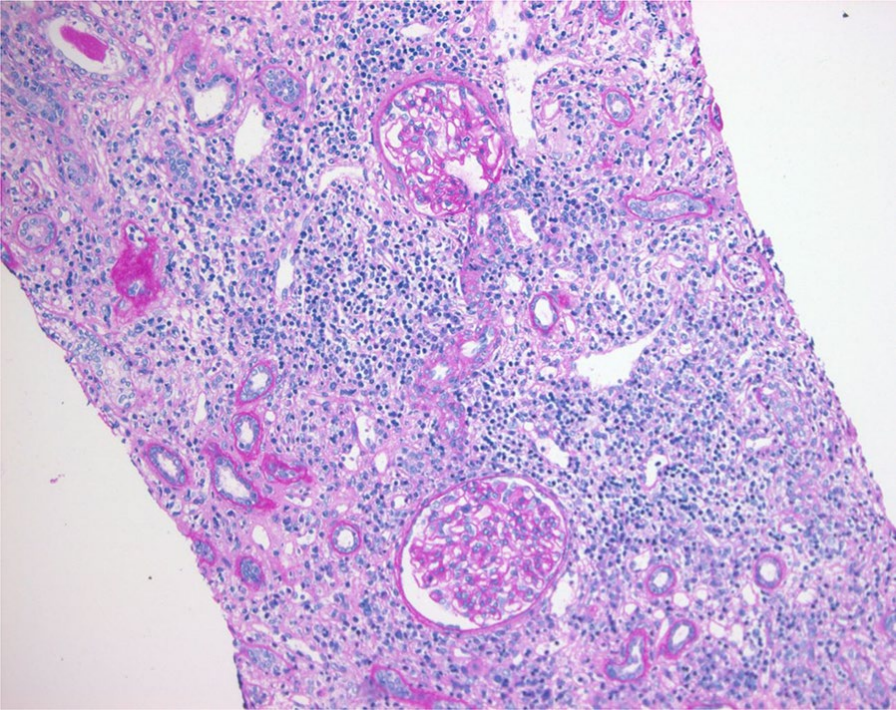
Light microscopic finding (haematoxylin and eosin stain ×200 high power field). Aggregation of lymphocytes, widely expanded interstitium, moderate interstitial fibrosis with mild tubular loss or atrophy

**Fig. 3:**
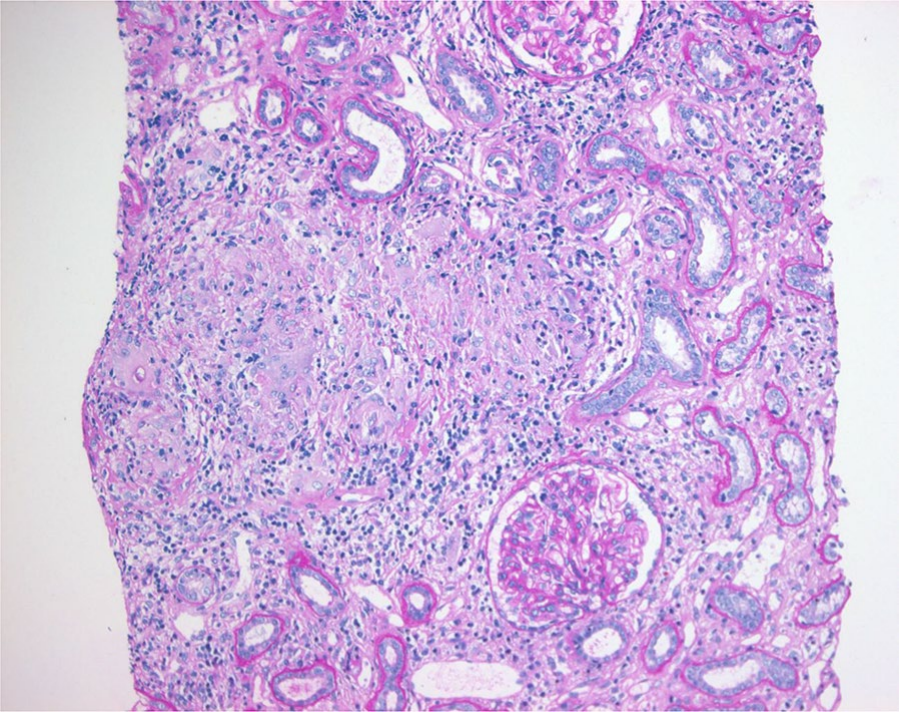
Light microscopic finding (haematoxylin and eosin stain ×200 high power field). Aggregation of epitheloid histiocytes, and multinucleated giant cells without necrosis

A 44-year-old male with complaints of nausea, vomiting, and weakness visited our hospital. He had suffered from back pain and had taken NSAIDs for 2 weeks from a local clinic about 1 month ago. He had no history of diabetes mellitus, hypertension, and familial renal diseases. In addition, there was no specific familial history about renal disease. On physical examination, he did not present with peripheral edema, and his lung sound on auscultation showed clear breathing sound without rale. He had no arthralgia and skin rash. His vital signs on admission were as follows: blood pressure, 120/80 mmHg; temperature, 36.9 °C; pulse, 90 beats per minute (bpm); and respiratory rate, 22 per minute. Laboratory findings on admission were as follows: hemoglobin 10 g/dL; white blood cells, 8 × 10^3^/µL; platelets, 200 × 10^3^/µL; eosinophil, 700/µL; blood urea nitrogen, 65 mg/dL; creatinine, 7.4 mg/dL; and total calcium, 9.7 mg/dL. Urinalysis showed protein +4, with >20 erythrocytes/high power field and urine calcium creatinine ratio was 32.81 mg/g. And the patient underwent 24-hour urine protein test and the amount of 24-hour urine protein was 741.2 mg/day. Serum levels of immunologic markers of the patient were shown as follows: complement 3 (C3), 97.4 mg/dL; complement 4 (C4), 27.0 mg/dL; perinuclear anti-neutrophil cytoplasmic antibody (pANCA), 1.1 U/mL; cytoplasmic anti-neutrophil cytoplasmic antibody (cANCA), 0.6 U/mL; anti-glomerular basement membrane antibody (anti-GBM Ab), <0.2 EU/mL. Chest radiograph and electrocardiograph were normal as well. Non-enhanced computed tomography showed mild enlarged kidney without other findings (Fig. 1). Initially, he received conservative treatment. His symptoms were not relieved and renal function was deteriorated despite of best supportive care. The patient underwent renal biopsy for an exact diagnosis and further management. Renal biopsy revealed segmental thickening of the glomerular basement membrane, mild segmental expansion of mesangium, aggregations of lymphocytes and epitheloid histocytes and multinucleated giant cells without necrosis in an expanded interstitium, and moderate interstitial fibrosis with mild tubular atrophy or loss. Immunofluorescent study demonstrated no deposition of immunoglobulins and complements in the glomeruli and tubulointerstitial regions (Fig. 2). Unfortunately, we could not see an eosinophilic infiltration on a light microscopic findings of interstitium and glomeruli, however, there was eosinophilia on initial laboratory examination. This pathologic findings such as interstitial infiltration of lymphocytes and moderate interstitial fibrosis accompanied by mild tubular loss or atrophy can explain a probability of tubular injury. The pathologic finding was also compatible with GIN (Fig. 3). Unfortunately we did not perform a stain for acid-fast bacilli on the biopsy specimens in order to rule out renal tuberculous infection. However, several laboratory examinations to differentiate tuberculosis and sarcoidosis that have histologic granulomatous lesions were performed. The serum level showed normal value of tuberculosis antigen-specific interferon-gamma assay (TB IFN-γ) and angiotensin converting enzyme. The levels of serum calcium and urine calcium execretion were also within normal range. Although a previous literature reported sarcoidosis induced GIN without extrarenal involvement, most patients with a GIN caused by sarcoidosis usually have extrarenal manifestations such as lung, skin, other organ involvement. In addition, unless there are no specific etiologies of AKI, we are good to look for etiologies of AKI elsewhere [4]. Therefore, this patient was suspected drug-induced acute granulomatous interstitial nephritis based on the recent history of NSAIDs use and normal findings of laboratory examinations. After the patient was put on 30 mg prednisolone, serum creatinine level began to decrease dramatically and reached 2.4 mg/dL.

## Conclusions

Acute interstitial nephritis is one of common causes of AKI. It is currently recognized that drugs are the most common cause of AIN. According to a recent report, drug-induced AIN is mainly caused by antimicrobials, proton pump inhibitors, and NSAIDs [5]. Acute granulomatous interstitial nephritis is a rare form of AIN. According to one report, GIN was <1 % of native renal biopsies. In addition, Bijol et al. [6] reported that GIN was mainly caused by drugs (44.7 %) and sarcoidosis (28.9 %). Granulomatous lesions consist of infectious granulomatous lesions and noninfectious granulomatous lesions. If histology of renal biopsy shows a granulomatous inflammation, we have to consider infectious disease including tuberculosis, and autoimmune/systemic disorders such as sarcoidosis and TINU syndrome. Less commonly, drugs can result in GIN. The implicated drugs include NSAIDs, antimicrobials, anticonvulsants, diuretics and occasionally allopurinol [7]. Early identification and withdrawal of causative drugs are most important for drug-induced AIN treatment. But if there is no clinical responsiveness after withdrawal of causative drugs, we can initiate treatment with corticosteroid [8]. However, there is no guideline for exact dose and duration of corticosteroid use in the patients with drug-induced AIN. In this case, although there were no eosinophilic infiltration, because accumulation of immunoglobulins and complements on the renal biopsy was shown and he had drug history of using NSAIDs, we could think that the GIN was related with drugs rather than other causes. According to case report of previous literature, clinical manifestations of GIN after using of NSAIDs varies from nephrotic-range proteinuria to vasculitis; however, NSAIDs induced interstitial nephritis is developed after months drug exposure and proteinuria of NSAIDs induced interstitial nerphritis is usually subnephrotic. Also, misdiagnosis of patients with acute GIN can result in end-stage renal disease [9]. Therefore, we initiated supportive care and tried to avoid exposure of nephrotoxic contents. Despite this effort, the serum creatinine continuously increased and his symptoms were not improved. So, the renal biopsy was immediately performed. The histopathologic findings such as granuloma and interstitial fibrosis and tubular atrophy usually indicate poor prognosis in acute tubulointerstitial nephritis. Therefore, prompt and proper management of acute granulomatous interstitial nephritis is very important in prognosis of AKI and can retard the progression to chronic kidney disease. There is no definite therapeutic trial for acute granulomatous interstitial nephritis yet, however, in recent retrospective study and case reports, treatment with moderate dosage of corticosteroid may be good option for acute granulomatous interstitial nephritis irrespective of etiologies [10]. Hence, we initiated moderate to high dose prednisolone and his serum creatinine decreased rapidly within a month. His uremic symptoms were all relieved after corticosteroid treatment. In conclusion, acute granulomatous interstitial nephritis is an extremely rare cause of renal failure. When AIN is strongly suspected, since its morbidity is significantly high, we should confirm diagnosis by means of renal biopsy and immediately initiate moderate to high dose prednisolone for acute granulomatous interstitial nephritis. In conclusion, we report a rare case, NSAIDs induced acute granulomatous interstitial nephritis. Although the GIN does not show good prognosis, an exact diagnosis and immediate treatment with moderate to high dose prednisolone will be helpful.

## Consent

Written informed consent was obtained from the patient for publication of this case report and any accompanying images.

## Abbreviations

AIN: acute interstitial nephritis
AKI: acute kidney injury
NSAIDs: nonsteroidal anti-inflammatory drugs
TINU: tubulointerstitial nephritis and uveitis
GIN: granulomatous interstitial nephritis

## References

[1] Praga M, González E (2010). Acute interstitial nephritis. Kidney Int.

[2] Krishnan N, Perazella MA (2015). Drug-induced acute interstitial nephritis: pathology, pathogenesis, and treatment. Iran J Kidney Dis.

[3] Joss N, Morris S, Young B, Geddes C (2007). Granulomatous interstitial nephritis. Clin J Am Soc Nephrol.

[4] Shivani S, Carter-Monroe N, Atta MG (2015). Granulomatous interstitial nephritis. Clin Kidney J.

[5] Muriithi AK, Leung N, Valeri AM, Cornell LD, Sethi S, Fidler ME (2014). Biopsy-proven acute interstitial nephritis, 1993–2011: a case series. Am J Kidney Dis.

[6] Bijol V, Mendez GP, Nosé V, Rennke HG (2006). Granulomatous interstitial nephritis: a clnicopathologic study of 46 cases from a single institution. Int J Surg Pathol.

[7] Inoue Y, Suga M (2008). Granulomatous diseases and pathogenic microorganism. Kekkaku.

[8] Praga M, Sevillano A, Auñón P, González E (2015). Changes in the aetiology, clinical presentation and management of acute interstitial nephritis, an increasingly common cause of acute kidney injury. Nephrol Dial Transplant.

[9] Schwarz A, Krause PH, Keller F, Offermann G, Mihatsch MJ (1988). Granulomatous interstitial nephritis after nonsteroidal anti-inflammatory drugs. Am J Nephrol.

[10] Ikeda A, Nagai S, Kitaichi M, Hayashi M, Hamada K, Shigematsu M (2001). Sarcoidosis with granulomatous interstitial nephritis: report of three cases. Intern Med.

